# Meeting report: 2017 Winter School on Therapeutic Ultrasound

**DOI:** 10.1186/s40349-018-0112-8

**Published:** 2018-06-06

**Authors:** Natasha D. Sheybani

**Affiliations:** Department of Biomedical Engineering, Health System, University of Virginia, Box 800759, Charlottesville, VA 22908 USA

**Keywords:** Therapeutic ultrasound, Focused ultrasound, Image guidance, Meeting

## Abstract

The Winter School on Therapeutic Ultrasound held its biennial meeting at the Ecole de Physique des Houches in Les Houches, France from March 26-31, 2017. The program brought together a globally and scientifically diverse group of faculty, clinicians, and trainees with interest in elements of therapeutic ultrasound. The meeting agenda was composed of daily lectures on various topics of relevance, supplemented with evening seminars, excursions, and networking opportunities. In anticipation of the next edition of the Winter School expected in 2019, we are nearly reaching the half way point between offerings of this unique and specialized meeting. Thus, this meeting report offers a reflection on the 2017 Winter School with the intention of setting the stage for 2019. Reviewed within this report are the lectures, student presentation series, and evening seminars encompassed within meeting agenda. It is my hope that this resource will be utilized by investigators within the therapeutic ultrasound community considering attending the Winter School in 2019.

## Introduction

The Winter School on Therapeutic Ultrasound is a unique workshop opportunity offered once every 2 years for students, postdocs, faculty, clinicians, and industrial partners to explore the rapidly expanding field of therapeutic ultrasound. This year’s workshop brought together individuals from multiple countries spanning North America, Europe, and the Middle East. Participants convened at the Ecole de Physique des Houches to engage in advanced multidisciplinary lectures provided by a globally renowned panel of invited speakers.

A typical day of the workshop consisted of 6-7 lectures, intermingled with opportunities to explore Les Houches and engage in activities such as skiing, hiking, walking, and networking with fellow attendees. The unique environment, located amid the breathtaking landscape of the French Alps, provided for a reserved and refreshing retreat wherein a diverse group of students and experts could engage about research in a relaxed, amicable, and approachable manner. Beyond daily lectures, additional learning opportunities included two special evening seminars, a documentary screening, and student presentations, all of which will be discussed in greater detail herein.

## Lectures

Each lecture was delivered by a world authority well-positioned to discuss a topic pertaining to therapeutic ultrasound within the context of his/her own research. The content of each lecture is summarized below.

### Acoustic propagation – Soft tissue

Robin Cleveland (University of Oxford) delivered a lecture on ultrasound propagation through soft tissue on behalf of Vera Khokhlova (University of Washington; Moscow State University). The core concepts of acoustics were overviewed in this lecture, including reflection, refraction, and impedance in the context of beam propagation, attenuation and power, sound speed and impedance. The content additionally advanced into topics such as nonlinearity, shock theory, KZK equation, Pennes Bioheat Transfer Equation, and heat deposition.

### Focusing ultrasound

Jean Francois-Aubry (CNRS; Institut Langevin) discussed the concept of wave focusing, techniques for numerical modeling of multi-transducer arrays, implications of sparse array spatial distribution for therapy, and the challenges of skull attenuation. Strategies for focusing and non-invasive target verification were elucidated, such as CT co-registry for skull characterization, single cavitation bubble induction at the focus to verify the target, radiation pressure detection by MRI, and exploitation of natural acoustic reflectors (e.g. kidney stones, microcalcifications) or systemically introduced acoustic amplifiers (e.g. targeted ultrasound contrast agents). Amid the progression towards non-invasive strategies for delivering therapeutic ultrasound to the brain, one of the major challenges for non-invasive transcranial therapy has been overcoming skull attenuation. Currently, the predominating algorithms for performing transcranial aberration correction are (1) reversal/phase shift correction, which optimizes energy deposition at the focus and (2) amplitude compensation, which optimizes pressure distribution within the focal plane. These techniques hold appeal for transcranial thermal and non-thermal therapies, respectively.

### Biophysics – Heating & thermometry

Gail ter Haar (Institute of Cancer Research, London) delivered a lecture on heating by ultrasound. The biological consequences of heating tissue are tunable based on temperature rise and duration. Induction of temperature rises ranging from 3 to 5 °C is generally classified as hyperthermia, while induction of temperature rises between 20 and 60 °C is categorized as thermal ablation. Monitoring of these temperatures via thermometry can occur in a variety of ways; strategies for thermometry include thermocouples, fiber-optic hydrophones (temperature and pressure feedback), MR thermometry, and ultrasound temperature mapping. This final strategy is an emerging technique whereby displacement serves as a proxy for heat induction within the tissue being imaged. Despite compelling advantages in the way of cost, availability, sensitivity, and precision, ultrasound thermometry faces limitations including lack of feedback regarding absolute temperature, poor sensitivity in the presence of fat, and motion artifacts.

### Cavitation – Applications

Larry Crum (University of Washington) introduced the topic of acoustic cavitation and passive cavitation detection, followed by a survey of emerging applications. The lecture delved into a myriad of applications that can extend from the biological consequences of cavitation, including sonothrombolysis, sonoporation and gene transfection, blood brain barrier disruption, and kidney stone comminution. Specific ultrasound techniques that harness acoustic cavitation to mediate these effects include histotripsy, as in the example of thrombolysis, and shock wave lithotripsy, as in the example of kidney stone fragmentation.

### Basics of ultrasound imaging

Thomas Deffieux (Institut Langevin, INSERM, ESPCI) provided an introduction to the principles of ultrasound imaging and how these concepts underlie the various modalities of ultrasound. Ultrasound is a versatile imaging modality that operates on the basis of sound wave transmission and reception. The defining principles of ultrasound imaging were discussed in the context of impedance, reflection, and transmission/reception of pulse echoes. The acoustic window and imaging applications for various transducer geometries - including linear array, convex (curved) array, and phased array – were introduced as a segue into subsets of ultrasound imaging, including B-mode, Doppler, ultrafast, and 3D imaging.

### Basics of Magnetic Resonance Imaging (MRI)

Florian Steinmeyer (Technische Hochschule Neurnberg) introduced the basic physics underlying nuclear magnetic resonance (NMR), including spin, magnetic moments, T1 and T2 relaxation, and induction decay. The translation from NMR to MRI was explicated in terms of pulse sequences, slice selection, phase encoding, and image reconstruction from complex magnetization. MRI is a powerful tool for imaging that, while slower than ultrasound with respect to scan time, holds particular appeal for high intensity focused ultrasound (HIFU) applications due to the capability for near real-time temperature mapping.

### Ultrasound transducers

Remi Berriet (Imasonic) delivered a lecture on medical ultrasound transducers, beginning with the main components of a transducer and the role of piezoelectric material in converting between electrical and mechanical energy. Essential characteristics of transducers include frequency, bandwidth/pulse length, and sensitivity. These characteristics extend to therapeutic transducers, but additional specifications that are of particular importance include focusing, mean power, and peak pressure. Ultrasound fulfills a variety of clinical needs, but design of the transducer can vary vastly depending on access requirements. An overview was provided of the considerations for external, endo-cavitary, and interstitial/intraluminal access using therapeutic ultrasound probes. Closing remarks were provided on safety criterion, safety evaluation, and ongoing developments such as improvement of efficiency, safety, and performance, reduction of treatment time, and optimization of coupling between imaging and therapy for improved targeting and monitoring of treatment.

### Monitoring & guidance – US

Mathieu Pernot (INSERM, Institut Langevin, ESPCI) discussed the importance of image guidance and monitoring for safety and efficacy of ultrasound therapy. Ultrasound enables image guidance for targeting, real-time monitoring, and post-treatment assessment. Pending exposure conditions, standard B-mode imaging can be useful for qualitatively detecting changes in tissue backscatter following treatment. However, emerging qualitative methods for detecting changes in tissue composition include ultrasonic thermometry and elastography. The former quantifies apparent displacement of sound scatterers to measure temperature as a function of strain. Limitations include influence of motion artifacts, necessity of a priori knowledge of tissue composition, and the fact that the dependence of the speed of sound on temperature is not monotonic for temperatures exceeding 50 °C. Soft tissues have been demonstrated to increase in stiffness with heating; thus, elastography quantifies changes in the stiffness of soft tissues as a proxy for temperature change. This modality is not as robust for heterogeneous/granular tissues, e.g. tumors, as compared with homogenous tissues.

### Monitoring & guidance – MRI

Bruno Quesson (Inserm, Universite de Bordeaux) delivered a lecture on MR monitoring and guidance. Tissue alteration depends on temperature and time of exposure, which on MRI monitoring, translates to mapping of thermal dose and temperature. Techniques for MR temperature mapping include quantitation of correlates such as diffusion, longitudinal relaxation, frequency shift of water resonance, and 1H spectroscopic imaging. MRI-guided ablation can be confounded by factors such as motion of mobile targets, cooling effect conferred by blood flow, and energy absorption by skin and bones. These factors must be overcome and monitored in order to ensure delivery of appropriate acoustic intensities at the focus. MRI techniques are varied in design and permit compensation for motion artifacts, monitoring of temperature elevation and/or tissue displacement, perfusion imaging, and in emerging applications, automatic feedback control of HIFU energy deposition.

### Tumor biology & physiology

Michael Horsman (Aarhus University Hospital) discussed biological mechanisms that extend from heat induction in the tumor microenvironment, and physiological considerations for effective application of hyperthermia to tumors - with emphasis on the role of vascular supply on tissue response. Studies have demonstrated at the cellular level that heat induction influences oxygen consumption, protein inactivation, and cell survival, among other effects. Tumor cell type, cell cycle, and microenvironmental parameters such as oxygenation, can also play a role in response to heating. Tumors are characterized by hypoxic microenvironments and aberrant vasculature, both of which are in part dictated by tumor size. Part of the challenge of heat induction in tumors is the heterogeneity in these characteristics and the resulting influences on heat deposition and damage. Pre-clinical studies are underway to explore how hyperthermia interfaces with different tumor microenvironments. Meanwhile, hyperthermia has already been demonstrated to be a potent enhancer of radiotherapy in patients, thereby implicating a role for therapeutic ultrasound in such combination therapies.

### Current applications of HIFU in oncology

Joo Ha Hwang (University of Washington) provided an overview of applications, both approved and emerging, of HIFU in oncology. Currently, the oncologic indications for HIFU include pain palliation and localized tumor ablation. In the example of pancreatic cancer, HIFU is used for pain control through the ablation of proximal nerves. A similar approach is used for palliation of painful bone metastases. Alternatively, HIFU has been used to ablate primary tumors as in the cases of prostate and liver cancers. For these applications, acoustic window is and will continue to be a critical feature of the instrumentation as HIFU becomes more widely adopted for focal therapy and palliation.

### Biophysics – Cavitation

Ronald Roy (University of Oxford) delivered a lecture on bubble acoustics, acoustic cavitation, and bubble dynamics. Bubbles are potent scatterers of sound. At low intensities, single bubbles respond as linear oscillators, mimicking the behavior of a classic 2nd order system and structure of a forced spring-mass-dashpot oscillator. The radius of spherical bubbles influences resonance frequency and scattering. In ultrasound imaging and therapy applications, systemically administered microbubbles serve as contrast agents. At high intensities, single bubbles can respond nonlinearly. In general, the response of bubbles to an acoustic pressure field is characterized by two modes of acoustic cavitation: stable and inertial. Stable cavitation refers to repetitive pulsations of the bubble about its spherical radius and is dominated by compressibility. Inertial cavitation, however, is mediated by liquid inertia and results in unstable growth that ultimately results to violent collapse. The physical effects of these modes of cavitation can include radiation stress, cavitation microstreaming, heating, collapse microjets, sonoluminescence, and radiation stress.

### Calibration & field characterization

Oleg Sapozhnikov (Moscow State University; University of Washington) provided insight into techniques for transducer calibration and acoustic field characterization. Techniques for observing an ultrasound field include indirect observation of effects such as radiation pressure, heating, or cavitation through a propagation medium, optical shadowgraphy (schlieren), and infrared emission from ultrasound-heated layers. Therapeutic ultrasound probes are typically characterized using a combination of approaches including acoustic pressure sensing (hydrophones), radiation force balance, calorimetry, laser vibrometry, and infrared imaging. In order to characterize the in situ field, however, it is necessary to know information about the source, medium, and modeling of wave propagation.

### The route to commercialization

Frederic Sottilini (CarThera) discussed the process of developing a product and achieving milestones towards commercialization in the context of CarThera’s experience. CarThera is a Paris-based company that pioneers ultrasound-based medical devices for the treatment of brain disorders. Thus far, its two breakthrough medical devices (Sonocloud© and Sonoprobe) span four indications, including Glioblastoma Multiforme, Alzheimer’s disease, brain metastasis of melanoma, and brain tumors. The pathway to commercialization involves identification of an unmet clinical need. The compelling need for minimally invasive interventions for the aforementioned neurological pathologies drives CarThera’s path towards commercialization. However, once a market opportunity for this need is confirmed, a multi-year process ensues wherein subsequent steps include comprehensive pre-clinical establishment, pre-clinical publications, regulatory tests, pilot clinical studies to establish safety and efficacy, and pivotal clinical studies. The achievement of these milestones ultimately predicates the review and approval of biomedical innovations for FDA designation (U.S.) and/or CE marking (Europe).

### Brain therapies – Delivery

Beat Werner (University Children’s Hospital Zurich) presented on the topic of brain applications of therapeutic ultrasound. Applications of MR-guided focused ultrasound (FUS) neurosurgery include thalamotomy against neuropathic pain, essential tremor, and Parkinson movement disorders; ablation of the anterior limb of the internal capsule for treatment of obsessive compulsive disorder (OCD); and brain tumor ablation. Functional neurosurgery and tumor ablation pose different challenges and criterion when it comes to focused ultrasound therapy. The acoustic environment, ablation rate, and treatment envelope pose unique challenges for effective tumor ablation despite its proven safety and therapeutic benefit in patients. Blood brain barrier opening with FUS and microbubbles is another category of therapy that is currently being explored in Phase I trials for brain tumors and early stage Alzheimer’s disease. The aforementioned applications have been enabled by the Insightec ExAblate MR-guided FUS system and CarThera SonoCloud.

### Histology for HIFU

Gail ter Haar (Institute of Cancer Research, London) discussed the basic types of tissue (muscle, nervous, connective, and epithelial) and strategies for analyzing them by histology. Histology is an essential technique for characterizing HIFU lesions at the cellular and tissue levels. Since HIFU is effectively a strategy for inducing cell death, the mode of histological analysis can be in part dictated by the nature of cell death (i.e. apoptosis versus necrosis). The sequence for tissue processing generally involves fixation, dehydration, infiltration and embedding, sectioning, mounting, staining, and analysis by microscopy. The most common histological stain is hematoxylin & eosin (H&E) staining, which stains for nucleic acids and other tissue components, respectively; this stain can be used to determine tissue integrity and identify regions of inflammation. A diverse range of immunohistochemical stains are available for evaluating more specific biological consequences of HIFU such as changes in perfusion, hypoxia, vessel patency, stress (e.g. heat shock proteins), progressive damage, inflammation, cell proliferation, etc.

### Ultrasound mediated drug delivery

Holger Gruell (University Hospital of Cologne, Germany) delivered a lecture on the use of ultrasound for drug delivery. In cancer therapy, standard chemotherapy delivery poses challenges including resistance, poor tumor uptake, off-target effects, stability, etc. Barriers to delivery include tight junctions between cells lining blood vessels that prevent extravasation into the tissue, renal filtration cutoff at which particles exceeding a certain size get taken up by liver and spleen, and more. Tumors are characterized by the enhanced permeability and retention effect, whereby leaky vasculature can facilitate uptake of nanoparticles, despite the presence of high interstitial fluid pressure. Ultrasound can mediate drug delivery by way of mechanisms such as sonoporation, cavitation, and hyperthermia, which can increase local vascular permeability, widening of junctions, transient pore induction, and even drug release in response to pressure and/or temperature changes.

### Thermal biology and thermal dose

Holger Gruell (University Hospital of Cologne, Germany) discussed thermal biology, reviewing its history and overviewing the biology of thermotoxicity as it pertains to cells, proteins, and membranes. The effects that extend from heating of these entities are dictated by thermal dose, a concept which was developed based on studies revealing a breakpoint in rate of cell survival (specifically a doubling in cell death) with every degree temperature increase above 43 °C. In general these effects can include increased metabolism, increased generation of reactive oxygen species, loss of clonogenicity, heat shock response, nuclear damage to proteins and DNA repair mechanisms, and ultimately, cell death. Below the threshold temperature, thermotolerance is likely to play a role in greater cell survival. However, the concept of thermal dose remains poorly characterized in vivo and in particular for temperatures above the supposed breakpoint of 43 °C. Meanwhile, thermotoxicity can have large variations across cell lines, species and microenvironments; thus, clinical hyperthermia has yet to be adopted as a mainstream technique despite demonstrated efficacy. Pre-clinical studies are still urgently needed to characterize this variability and to delineate differences between normal and tumor cell response to heating given the emerging role of immune response to high intensity therapeutic ultrasound application.

### Competing technologies

Afshin Gangi (University Hospital Strasbourg France; Kings College London) provided examples of competing technologies, overviewing the various imaging techniques available for image guidance of focal therapies, including MRI, PET, and ultrasound. Subsequently, clinical case studies were shared to highlight the applications of non-invasive or minimally invasive modalities beyond HIFU that are available for pain palliation and tumor decompression, as in the examples of cryoablation and RF ablation. The conclusion from these highlights was that in the emerging era of personalized medicine, each intervention is likely to have its own place in an interventional radiologist’s arsenal for cancer therapy. A final highlight of the lecture was the potential role of the abscopal effect in adaptive immunity that can stem from these interventions; this compelling prospect has been elucidated by multiple clinical observations wherein ablative treatment of a primary tumor site has led to shrinkage of distant, untreated lesions.

### Histotripsy

Oleg Sapozhnikov (Moscow State University; University of Washington) presented a lecture on histotripsy on behalf of Vera Khokhlova (University of Washington; Moscow State University). In contrast to thermal ablation, histotripsy is a method of mechanical ablation whereby tissue is disintegrated using high intensity, pulsed energy that leads to cavitation and shock wave formation. There are two modes of histotripsy: (1) cavitation cloud (short, microsecond-scale pulses) and (2) boiling (long, millisecond-scale pulses). In this lecture, the physics and instrumentation underlying each of these modes was covered in detail. Histotripsy results in mechanical tissue fractionation with sharp lesion margins and is detectable by various modalities, such as histology, ultrasound imaging, MR imaging, and shear wave imaging. Histotripsy has been demonstrated to be efficacious in a variety of applications spanning urology, cardiology, gastroenterology, cancer therapy, immunomodulation, and more.

### Tumor immunology/immunotherapy

Elizabeth Repasky (Roswell Park Cancer Institute) provided an overview of immunology and the implications of cancer immunotherapy for ultrasound applications. The two major branches of adaptive immune response are humoral immunity (involving antibody production and B lymphocyte mediated response) and cell-mediated immunity (involving production of a host of cell types, cytokines, and T lymphocyte mediated response). These responses are generally driven by the presence of antigens. In the setting of a tumor, the presence of tumor antigens drives recognition of, and response to, tumor by antigen-presenting cells and T cells in the tumor and draining lymph node. Tumors are capable of interfering with this sequence of cancer immunity through checkpoint molecules, wherein tumor cells hijack key checkpoints on which immune cells typically rely to avoid over-acting on healthy cells. In doing so, tumor cells can evade the immune system. Checkpoint inhibitor molecules can block the interaction between T cells and tumor cells to effectively lift the veil of tumor immunosuppression. Anti-PD1 and anti-CTLA4 therapies have already found success in the clinic. The coupling of these interventions with focal therapies that can induce immunogenic tumor cell death - e.g. chemotherapy, radiation therapy, thermal therapy, HIFU, cryotherapy – holds promise for bolstering antitumor immune response.

### Lithotripsy & Shock Wave Lithotripsy (SWL)

Robin Cleveland (University of Oxford) discussed the development of extracorporeal SWL. The lecture began with an introduction to the history of SWL, description of what constitutes a shock wave, and key physics underlying SWL such as stress wave formation and cavitation bubble dynamics. SWL has revolutionized the treatment of kidney stones as a mainstream therapy since its FDA approval in 1984. However, SWL has also been linked with injury, including hematuria, subcapsular hematomas, onset hypertension, and anecdotally, diabetes mellitus. Despite the advancement of competing technologies such as ureteroscopy and percutaneous nephrolithotomy, SWL still remains a major treatment modality. Emerging applications include chronic soft tissue pain palliation and repair, scar tissue removal and osteogenesis by bone tissue disruption.

### Matching transducer geometry to clinical targets

Cyril Lafon (LabTau, INSERM; University of Virginia; FUS Foundation) delivered a lecture on design of ultrasonic devices for conformal therapies. The nature of clinical targets often dictates the requirements for beam shape and desired bioeffects. In order to modulate these parameters extracorporeally, it is necessary to consider the transducer frequency, transducer diameter, exposure conditions such as power and duration, and tissue composition effects leading to attenuation. Examples of translational efforts that have led to clinical devices for conformal ultrasound treatment include a disposable non-image guided device for HIFU-induced cyclo-coagulation in the treatment of refractory glaucoma (EyeTechCare) and the SonoCloud implantable ultrasound device for repeated BBB-opening (CarThera). The remainder of the lecture focused on the preclinical and clinical efforts that led to the development and implementation of these devices.

### Experimental design

Gail ter Haar (Institute of Cancer Research, London) provided an overview of methods for properly designing experiments to answer scientific questions pertaining to therapeutic ultrasound. The four classic experimental models are in vitro (cells), ex vivo (excised tissue), in vivo (animals), and in silico (computational model). Guidance was provided on how to select an appropriate model from among these options, the importance of ultrasound field calibration, and criterion for reporting exposure conditions.

Jean Francois-Aubry (CNRS; Institut Langevin) closed the winter school lecture series with an overview of FDA and CE approved devices for therapeutic ultrasound.

## Special events

### Documentary screening

Filmmaker Martin Freeth showcased a short film following a young patient who underwent treatment for essential tremor with MRI-guided FUS. Afterwards a longer documentary describing The Antikythera Mechanism was aired. This documentary described researchers’ discovery and journey to unlocking the complexities of the first known analogue computer using gamma-ray and high-resolution X-ray tomography. A 5-min trailer for this documentary, entited “The X-Ray Time Machine” is available here.

### Evening lectures

Two special evening lectures were offered in order to provide students with yet another opportunity to engage with the faculty on topics that piqued there curiosities. The first in this series was led by Florian Steinmeyer and Bruno Quesson and entitled “What you always wanted to know about MRI and did not dare to ask.” In this session, students formulated a list of questions pertaining to the basics of MRI imaging, MRI applications, types of MRI imaging, challenges of imaging in specific applications, etc. to which the faculty provided detailed answers.

Larry Crum delivered the Gluhwein Lecture on “Trials and Tribulations of Translation,” wherein he discussed his experiences with translating ultrasound technologies from bench to bedside. The lecture provided a candid glimpse into the nuances of commercializing biomedical innovations – e.g. financial investments, patents, intellectual property, market identification, etc. – and the ways in which translational efforts can succeed and fail. The lecture closed with practical advice for students based on the lessons that Dr. Crum learned from his experiences.

### Student presentations

Over 40 participants, including graduate students, post-doctoral fellows, and members of industry, each gave a three-minute oral presentation highlighting their ongoing research followed by a two-minute Q&A session. Awards were given for the top three presenters, honorable mentions, best overall presentation, and best questions.

The Winter School on Therapeutic Ultrasound 2017 was directed by Gail ter Harr (Sutton), Vera Khokhlova (Moscow) and Jean-Francois Aubry (Paris), and organized by Thomas Deffieux (Paris), Cyril Lafon (Lyon) and David Melodelima (Lyon). The next Winter School on Therapeutic Ultrasound will be held in Spring 2019 in Les Houches, France.

## Abbreviations

FUS: Focused ultrasound
H&E: Hematoxylin & eosin
HIFU: High intensity focused ultrasound
MRI: Magnetic resonance imaging
NMR: Nuclear magnetic resonance;
OCD: Obsessive compulsive disoreder
SWL: Shock wave lithotripsy

